# Roost selection by synanthropic bats in rural Kenya: implications for human–wildlife conflict and zoonotic pathogen spillover

**DOI:** 10.1098/rsos.230578

**Published:** 2023-09-13

**Authors:** Reilly T. Jackson, Paul W. Webala, Joseph G. Ogola, Tamika J. Lunn, Kristian M. Forbes

**Affiliations:** ^1^ Department of Biological Sciences, University of Arkansas, Fayetteville, AR 72701-4002, USA; ^2^ Department of Forestry and Wildlife Management, Maasai Mara University, Narok, Kenya; ^3^ Department of Medical Microbiology, University of Nairobi, Nairobi, Kenya

**Keywords:** human–bat contact, human–wildlife interface, Megadermatidae, Molossidae, urbanization, wildlife conservation

## Abstract

Many wildlife species are synanthropic and use structures built by humans, creating a high-risk interface for human–wildlife conflict and zoonotic pathogen spillover. However, studies that investigate features of urbanizing areas that attract or repel wildlife are currently lacking. We surveyed 85 buildings used by bats and 172 neighbouring buildings unused by bats (controls) in southeastern Kenya during 2021 and 2022 and evaluated the role of microclimate and structural attributes in building selection. We identified eight bat species using buildings, with over 25% of building roosts used concurrently by multiple species. Bats selected taller cement-walled buildings with higher water vapour pressure and lower presence of permanent human occupants. However, roost selection criteria differed across the most common bat species: molossids selected structures like those identified by our main dataset whereas *Cardioderma cor* selected buildings with lower presence of permanent human occupants. Our results show that roost selection of synanthropic bat species is based on specific buildings attributes. Further, selection criteria that facilitate bat use of buildings are not homogeneous across species. These results provide information on the general mechanisms of bat–human contact in rural settings, as well as specific information on roost selection for synanthropic bats in urbanizing Africa.

## 1. Introduction

Urbanization has a pervasive and continuing impact on wildlife species. While urbanization has led to habitat loss for wildlife, some species use environmental conditions and structures that are products of human presence [1–4]. Many wildlife species within the urban human–wildlife interface are known to harbour zoonotic pathogens, and this close connectivity can facilitate pathogen spread (spillover) and spillback of these pathogens between wildlife and humans [5–7]. Close interactions often also cause human–wildlife conflict which can lead to human-induced wildlife mortality [8]. Information to inform contact- and conflict-mitigation strategies is needed to improve outcomes for both groups and is a key priority in the growing field of urban ecology.

Model systems that identify and characterize regions of high-risk human–wildlife interfaces will be critical to efforts to reduce human–wildlife conflict and pathogen spillover. Bats are progressively inhabiting urbanizing landscapes, with some species increasingly adapting and exploiting resources in these regions, while others may be moving into those areas due to anthropogenic land-use changes that destroy native habitat [9–12]. Some species are known to harbour pathogens, especially viruses, that can spill over into humans and domestic animals [13,14]. Bats are also increasingly imperiled, with 15% of bat species considered endangered or threatened, primarily due to anthropogenic actions such as land-use change, hunting, persecution and urbanization [11]. They provide several critical ecosystem services, including insect suppression, seed dispersal and pollination of many tropical plants, and addressing their conservation needs is important to environmental function [15,16]. The increasing use of human areas, such as buildings, connects bats to people and domestic animals intimately, thus creating opportunities for pathogen spillover, spillback and bat mortality at this cross-section of the human–wildlife interface [16,17].

Bats have been recorded in urban areas on all human-inhabited continents, and often roost in human structures such as houses, schools, offices, abandoned buildings and bridges [16,18]. For bats, human structures can provide stable shelter, breeding sites and safety from predators [19,20]. Structural attributes of buildings, such as their height, age, complexity and composition have been reported as important for determining bat roost suitability [19,21]. However, studies on this topic usually suffer from key limitations, often due to practical necessities while conducting research on bats in difficult environments, including low roost sample numbers, missing or inadequate controls, and statistical approaches that fail to account for interactions among relevant covariates (e.g. [22,23]). These limitations ultimately impair the ability to draw inferences that can inform strategies to mitigate human contact with wildlife. Further, the global ubiquity of synanthropic bats, their potential to host zoonotic pathogens, and their conservation importance make them key systems to understand how human–wildlife contact occurs and can be mitigated in rural and urbanizing settings [13–15,24].

Accounting for interactions among relevant covariates and the mediated effect of variables is necessary for understanding nuances of habitat selection by wildlife. Use of structural equation models (SEMs) is a method that allows for comparison of multiple covariates, but also can identify both direct and indirect interactions among variables [25]. This method allows for the *a priori* formulation of hypothesized causal models based on previous empirical research and calculates partial regression coefficients corresponding to predicted causal effects. By developing models using the existing literature on roost selection, SEMs can identify covariates with direct effects on roost selection, as well as variables that may indirectly impact (but are still important) selection criteria through their impact on direct effects. Fine-scale identification of these features can provide a comprehensive characterization of structures facilitating human–wildlife contact.

We investigated the features of buildings that contribute to bat roost use in rural southeastern Kenya to identify the traits of anthropogenic structures that bats select. This region has several bat species known to host a variety of pathogens and that roost in buildings with humans and domestic animals [10,26–28]. By understanding building characteristics key for bat roost selection, we can better characterize hotspots of human–wildlife contact in urbanizing areas and provide information to help target measures that mitigate contact between bats and humans. We applied SEMs to understand the direct and indirect interactions among building features, microclimate, and bat use. These data contribute important information in an area of the Global South where human contact with bats and disease risks are high, but where data are scarce. More broadly, we fill a need for information on bat roost selection globally and provide a model system for understanding human–wildlife contact in progressively urbanizing areas.

## 2. Methods

### 2.1. Study area

This study was conducted in Taita-Taveta County, in rural Kenya, during August–October 2021 and January–April 2022 (electronic supplementary material, figure S1). The region covers an approximate area of 17 000 km^2^ and is characterized by high diversity of wildlife, with patches of isolated cloud forest between 1400 and 2200 m above sea level surrounded by lower-elevation (400 to 1400 m above sea level) grasslands and woodlands, all distributed amongst rural villages and smallholder farms [29,30]. The climate is semi-arid and has an average annual temperature of 23°C [31,32]. Typically, there have been two rainy seasons, March to May/June and October to December, with an average yearly rainfall of 150 to 600 mm in the lowlands and 800 to 1200 mm in the highlands [31,32]. However, an ongoing drought has reduced the rains since 2019 [33]. Urbanization has dramatically increased over recent decades in Taita-Taveta County, with over 95% of original forest cover cleared and a 700% increase in development landcover [34,35]. Bat diversity is high here, with over 30 species recorded in the county [36] and humans often have bats living in their buildings (electronic supplementary material, figure S2). The region has also been identified as a hotspot for zoonotic disease emergence and numerous bat species in the area are known to host a diversity of pathogens [27,28,37].

### 2.2. Data collection

We identified buildings used by bats via word-of-mouth conversations with community members throughout the study area. Roost coordinates and elevation were recorded using a handheld GPS unit (model: GPSMAP 64sx; Garmin International Ltd, Olathe, KS, USA), and upon permission from homeowners and care takers, we entered buildings to confirm active bat presence and species identity. Bats were captured from buildings using hand nets at roosts or mist nets placed at exit points and identified to species level based on Patterson & Webala [38]. We identified slit-faced bats (Family: Nycteridae) to genus only because the taxonomy of this genus is complicated and requires revision, reducing our ability to correctly identify to species [18,39].

For every bat-occupied building, we selected at least two of the closest accessible buildings that had both no sustained (greater than 1 night/day) bat use within the homeowner's memory and no physical evidence of bat use (e.g. guano deposits, urine staining, bat-associated smells), as controls for microclimate and structural attribute comparison. We collected the following information from each roost type (used and unused by bats): (1) maximum daily light (lux), maximum daily temperature (°C) and maximum daily relative humidity (rH) (measured with an Enviro-Meter, ThermoFisher Scientific, Watham, MA); (2) building height from base to peak of the structure (m); (3) building aspect (cardinal direction of bat entry into building); (4) roof material; (5) wall material (categorized as cement, mud or other (metal, stone, unknown brick type)); (6) presence or absence of compartment above ceiling of the main living area (i.e. attic); and (7) presence/absence of permanent human occupants in the building (electronic supplementary material, S2). Microclimate data were collected between 11.00 and 17.00 based on preliminary data collection showing the highest daily temperatures during this period (electronic supplementary material, table S1). Microclimate characteristics and building aspect were measured at the roost in bat-occupied buildings; in control buildings, these characteristics were measured at equivalent roost positions (i.e. where bats were roosting in similar structures) [40].

Building variables were assessed for collinearity using Spearman's correlation coefficient, although we detected no correlation among numeric variables (‘corr’ in ‘survival’ R package [41]). Two variables, roof material and ceiling presence, showed no variation among surveyed buildings (greater than 80% of surveyed buildings had the same characteristic) and were dropped from analyses (electronic supplementary material, table S2). Aspect was not included in analyses due to most buildings having multiple entry points used by bats (electronic supplementary material, table S2). Instead of using raw relative humidity levels, due to the inherent relationship with temperature [42], we used humidity and temperature data collected at buildings to calculate water vapour pressure (WVP; kPa) and used this as a measurement for moisture content at roosts [43,44].

### 2.3. Statistical analyses

**Figure 1.**
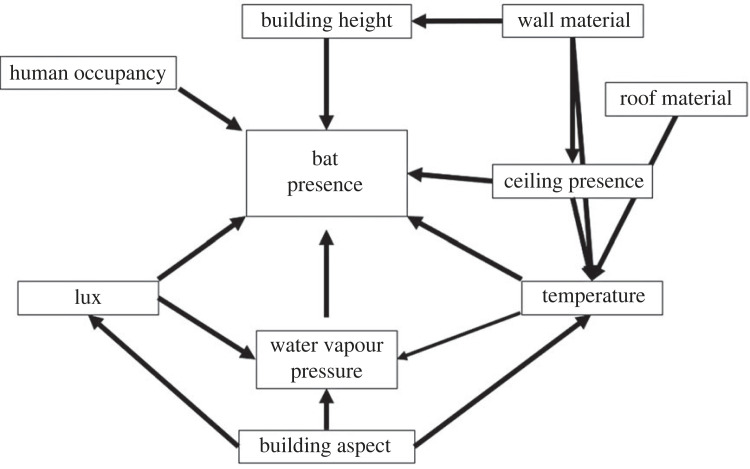
We used a pre-conceived conceptual model containing all measured structural and microclimate features to guide structural equation model (SEM) development for assessing variables important to building roost selection by bats. Pathways were conceived based on knowledge of bat ecology, the impacts of different structural features on microclimate, and building construction limitations.

Prior to analysis we tested whether the grouping of sites (i.e. selection of one occupied building and the two closest non-occupied buildings as controls) had any effect on building selection (‘lme4’ R package) [45]. Groupings did not have a significant effect on results, and so were not included in model structures (electronic supplementary material, table S3). We used SEMs to evaluate direct and indirect effects of building features on bat roost selection (‘lavaan’ R package) [46]. Direct effects are covariates with an unmediated impact on response variables whereas indirect effects are covariates mediated through another covariate. Model structure was guided by a pre-conceived conceptual model containing all measured building features (figure 1). To understand the importance of different factors to bat species, we further divided our main dataset into two groups: buildings used by co-roosting molossid bats (*Mops condylurus* and *M. pumilus*, formerly *Chaerephon pumilus*) and those used by the regionally endemic megadermatid, *Cardioderma cor*. We used SEMs to evaluate buildings used by molossids; however, due to fewer data points, we used different methods to analyse building roost selection by *C. cor* (described below).

For our all species and molossid datasets, analysed via SEMs, we used diagonally weighted least-squares (DWLS) estimation, with a Bollen-stine bootstrap resampling procedure to estimate standard errors (1000 bootstrap samples). This approach is robust to non-continuous response variables [25,47]. Model fit was evaluated using the *χ*^2^ statistic, comparative fit index (CFI) and the root mean square error of approximation (RMSEA) [25]. Both the *χ*^2^ statistic and RMSEA values are absolute fit indices which determine how well the *a priori* model structure fits the data. A non-significant (*p* > 0.05) *χ*^2^ statistic and significant RMSEA (*p* ≤ 0.05) indicate good model fit. CFI is a relative fit index, which assesses the relative improvement in fit of the *a priori* model structure compared to a baseline model, with CFI ≥ 0.9 indicating acceptable fit. Only models that fitted sufficiently proceeded to further interpretation. The final SEMs for our main dataset included direct effects on bat roost selection from maximum daily light (lux), WVP, temperature, the presence or absence of permanent human occupants, and building height, and indirect effects from wall material moderated through its influence on building height.

**Figure 2.**
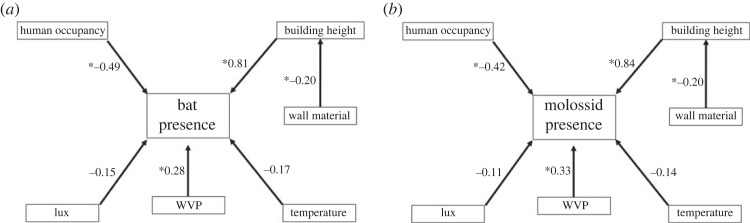
Relationships of microclimate and structural attributes on bat selection of building roosts for (*a*) buildings used by all bat species and (*b*) buildings used only by molossid bats (*Mops condylurus* and *M. pumilus*) analysed with structural equation models. Numbers adjacent to arrows are the total path regression coefficients for each variable's effect on bat presence. The greater the absolute value of the number, the larger the impact on bat presence. Asterisks (*) preceding each regression coefficient represent statistically significant relationships between variables (*p* < 0.05).

As a general guide, an SEM requires at least five rows of data per path coefficient [48], meaning at least 40 buildings were required per species for our model structure. We identified fewer buildings used by *C. cor* (*n* = 18 used buildings, 36 associated control buildings), which prohibited the use of SEMs. Therefore, for this species we used generalized linear models (GLMs) with a binomial error structure and logit link function to assess the role of microclimate and structural attributes in building selection (‘glm’ in R base package). The GLM structures were guided by the pre-conceived conceptual model, as in SEMs, with a stepwise regression approach to identify important variables from the main covariates: building height, human occupancy, lux, temperature, WVP, and wall material. Model fit of GLMs was checked with *χ*^2^ goodness-of-fit tests [49]. We identified the best performing model(s) with Akaike information criterion corrected for small sample size (AICc) scores. Models within 2 AICc units were considered competitive, and the weighted averages of coefficients were calculated for equally competitive models (R package ‘MuMIn’) [50]. All analyses were conducted in RStudio v. 2022.7.1 + 554.

## 3. Results

We identified 85 buildings used by bats and 172 nearby control buildings. Bats inhabited homes with humans (*n* = 31), abandoned buildings (*n* = 15), schools *(n* = 11), offices (*n =* 6), storage buildings (*n* = 5), staff lodging buildings (*n* = 5), isolated kitchen buildings (*n* = 3), medical dispensaries (*n* = 2), guest houses (*n* = 2) and conference buildings (*n* = 2). We also found bats roosting in one boma (a fortified livestock enclosure), one church, and one shop. Bats roosted inside buildings, mostly at the junction of the walls and roof (*n* = 37), the seam of the roof (*n* = 30), along roof structural support beams (*n* = 23), or under furniture (*n* = 2). Roost elevation ranged from 536 m a.s.l. to 1439 m a.s.l. (*x* = 952.42 ± 20.09 m a.s.l.) and we present the first records of *C. cor* presence above 940 m a.s.l. (902.72 ± 52.24 m a.s.l.; range: 536–1135 m a.s.l.).

Eight bat species were found roosting inside human structures (table 1). Over 25% of bat-occupied buildings contained more than one bat species roosting within the building (*n* = 22; table 1). *Mops pumilus*, the most frequently encountered species (*n* roosts = 65), co-roosted with six different species including *Epomophorus wahlbergi* (*n* = 1); *C. cor* (*n* = 2); *M. condylurus* (*n* = 13); *Nycteris* sp. (*n* = 1), *Rhinolophus cf. lobatus* (*n* = 2); and *Scotophilus andrewreborii* (*n* = 2) (table 1). One building contained three species co-roosting together (*C. cor*, *M. pumilus* and *R. landeri*). Nonvolant young of *C. cor* were observed in roosts in October and March–April, whereas young of *M. pumilus* and *M. condylurus* were observed during February–April. Nonvolant young of a slit-faced bat (*Nycteris* sp.) were observed in February. Nonvolant young of the other species were not observed.

**Table 1.** Eight bat species were found roosting in human buildings during surveys throughout communities in Taita-Taveta County, Kenya, 2021–2022. The number of other species sharing roosts refers to the number of other bat species a focal species was found roosting with across all shared buildings. All species except *Hipposideros caffer* were documented sharing buildings with at least one other bat species.

species	total no. of building roosts	no. of roosts shared with other species	no. of other species sharing roosts
*Cardioderma cor*	18	4	2
*Epomophorus wahlbergi*	1	1	1
*Hipposideros caffer*	1	0	0
*Mops condylurus*	13	13	1
*Mops pumilus*	65	21	6
*Nycteris* sp*.*	4	1	1
*Rhinolophus cf. lobatus*	3	3	2
*Scotophilus andrewrebori*	3	2	1

### 3.1. Buildings used by any bat species

**Figure 3.**
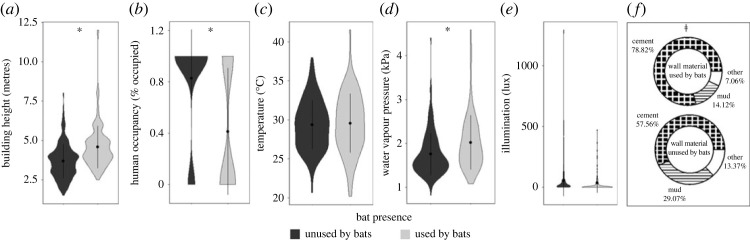
Comparison of microclimate and structural attributes that contributed to selection of buildings as roosts by any bat species: (*a*) building height; (*b*) human occupancy; (*c*) building temperature; (*d*) building water vapour pressure; (*e*) illumination; (*f*) wall material. Asterisk (*) and alveolar clicks (≠) denote statistically significant (i.e. *p* < 0.05) direct and indirect effects, respectively, of covariates on bat roost selection identified via structural equation models. Dots and bars inside violins represent mean and standard deviation of the raw data, respectively.

In our all-species SEM, model fit was validated by all but one metric (*χ*^2^ = 0.06, CFI = 0.99, RMSEA = 0.06, *R*^2^ = 0.99). The model showed that height and WVP had significant, direct positive effects on bat selection of buildings, with bats selecting taller buildings with higher WVP compared to controls (*p* < 0.01). The presence of permanent human occupants had a significant, direct negative effect, with bats selecting buildings without permanent human occupants compared to controls (*p* ≤ 0.03; figures 2*a* and 3; electronic supplementary material, tables S4 and S5). Wall material had a significant indirect effect mediated through building height (*p* < 0.01; figure 2*a*), with cement-walled buildings being taller (mean = 4.79 ± 0.09 m) than mud (mean = 3.67 ± 0.18 m) or other material buildings (mean = 4.25 ± 0.45 m), having a positive impact on bat presence.

### 3.2. Buildings used by molossid bats

**Figure 4.**
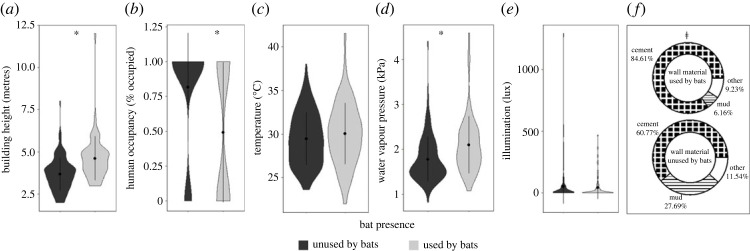
Comparison of microclimate and structural attributes that contributed to selection of buildings as roosts by molossid bat species: (*a*) building height; (*b*) human occupancy; (*c*) building temperature; (*d*) building water vapour pressure; (*e*) illumination; (*f*) wall material. Asterisk (*) and alveolar clicks (≠) denote statistically significant (i.e. *p* < 0.05) direct and indirect effects, respectively, of covariates on bat roost selection identified via structural equation models. Dots and bars inside violins represent mean and standard deviation of the raw data, respectively.

We identified 65 buildings used by molossids and surveyed 130 associated control buildings. For our molossid-only SEM (*χ*^2^ = 0.29, CFI = 0.99, RMSEA = 0.04, *R*^2^ = 0.98), building height and WVP had similarly significant direct positive effects on bat use of buildings, whereas presence of permanent human occupants had a significant direct negative effect (*p* ≤ 0.03; figures 2*b* and 4; electronic supplementary material, tables S6 and S7). Wall material had a significant indirect effect, mediated through building height (*p* < 0.01; figure 2*b*; electronic supplementary material, table S6), with cement-walled buildings being taller (mean = 4.71 ± 0.18 m) than mud (mean = 3.87 ± 0.31 m) or other material buildings (mean = 4.25 ± 0.46 m), having a positive impact on bat presence.

### 3.3. Buildings used by *Cardioderma cor*

**Figure 5.**
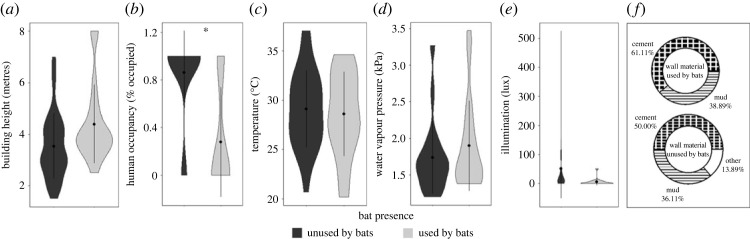
Comparison of microclimate and structural attributes that contributed to selection of buildings as roosts by *Cardioderma cor*: (*a*) building height; (*b*) human occupancy; (*c*) building temperature; (*d*) building water vapour pressure; (*e*) illumination; (*f*) wall material. Asterisk (*) denotes statistically significant differences (i.e. *p* < 0.05) based on generalized linear models used to analyse data. Dots and bars inside violins represent mean and standard deviation of the raw data, respectively.

We identified 18 buildings used by *Cardioderma cor* and surveyed 36 associated control buildings. We derived six equally ranked GLMs explaining building roost selection by *C. cor* (table 2). Averaged models showed *C. cor* selected buildings that were less likely to have permanent human occupants (*β* = −2.80; *p* < 0.01) when compared to control buildings (figure 5; electronic supplementary material, table S8).

**Table 2.** Rank of generalized linear models used to investigate the impacts of six covariates on roost selection of buildings by *Cardioderma cor*. Models were selected for comparison based on Aikake's information criterion corrected for small sample sizes (AICc) score and models within 2 ΔAICc units were considered competitive, with the weighted averages (*W_i_*) of coefficients calculated for equally competitive models. We present the number of coefficients in each model (*k*) and results from χ-square goodness-of-fit-tests (*χ*^2^).

model	AICc	ΔAICc	*W_i_*	*k*	*χ*^2^
building height + human occupancy + lux	48.78	0	0.30	4	0.84
human occupancy + lux	49.72	0.94	0.19	3	0.92
building height + human occupancy + lux + temperature + water vapour pressure + wall material	50.34	1.55	0.14	7	0.83
building height + lux + human occupancy + water vapour pressure	50.47	1.68	0.13	5	0.84
building height + human occupancy + wall material	50.57	1.78	0.12	4	0.84
building height + human occupancy + lux + temperature	50.78	2.00	0.11	5	0.77

## 4. Discussion

We use bats, highly pervasive taxa in anthropogenic environments, as a model system to understand features of urban settings that attract and repel wildlife species. Our study demonstrates that building roosts of synanthropic bats in rural East Africa are associated with specific microclimate and structural attributes. Bats in general selected buildings that were taller, had higher WVP, and had lower presence of permanent human occupants compared to control buildings. Building roost selection was moderated by the effect of wall material on building height, with cement-walled buildings being taller than buildings made with other materials. However, our models had trouble incorporating data from the two disparate taxa into one model. When separated by taxonomic group, molossids selected building roosts based on reduced presence of permanent human occupants, building height, WVP, and wall material moderated by building height. By contrast, although our *Cardioderma cor* sample size was smaller than ideal, we found that this species focused on the absence of permanent human occupants in their selection criteria. These results provide valuable general information on synanthropic bat roost selection and help characterize contact between humans and bats in an urbanizing region of the Global South.

Building features that increase the likelihood of bat use can have major ramifications for human–wildlife contact and conflict. We identified direct and indirect pathways influencing bat building use, with models showing direct positive effects of building height and WVP and direct negative effects of presence of human occupants on bat use of buildings. Height is important for wildlife to avoid ground-based predators and also bat species that must take off from tall roosts to become airborne [51,52]. Avoidance of buildings with permanent human occupants is a likely result of reduced disturbance and predation risk since many homeowners in this region have reported killing bats in their residence [53]. We also identified a significant indirect effect of wall material on bat use of buildings, mediated through the significant effect of building height. Although this has not been cited previously as an important characteristic in building roost selection by bats, wall material is a crucial factor in building height as certain materials, like cement, provide structural integrity to increase building height. While previous studies may have missed the effects of wall material due to counteracting impacts, SEMs have the power to identify these intricacies and will be important for future research on similar questions [25].

Our large dataset enabled us to compare building preferences between taxa, whereas previous investigations of building roost selection by bats have only focused on a single species or have grouped multiple species together [19,40]. Our results highlight the limitation of combining disparate bat species in analyses, showing that not all synanthropic species are homogeneous in their roost preferences and that they may present different risks for contact with humans. For example, within our dataset most buildings selected by *C. cor* had no permanent human occupants. In comparison, almost half of buildings selected by molossids had permanent human occupants, which could put them at higher risk for contact with humans. Moreover, given that bats are present on all human-inhabited continents and have been recorded roosting in buildings throughout the world [16], this principle is likely to be relevant in other settings.

We observed numerous cases of roost sharing by multiple bat species. While roost use by multiple species is not uncommon in bats, large multi-species aggregations may play an important role in viral evolution and diversity within bat populations [54–58]. Currently, bat species documented to roost in multi-species assemblages have largely been described to co-roost with 2–3 other bat species [59,60]. We found that *M. pumilus* roosted with at least six other bat species in anthropogenic roosts, indicating great potential for multi-species interactions. In this region, *M. pumilus* hosts a high diversity of viruses, including coronaviruses, flaviviruses, adenoviruses and potentially filoviruses [26,28,58,61]. Several of these viral families are highly capable of recombination, meaning that these diverse roosts may facilitate viral evolution in anthropogenic environments [62]. Given that *M. pumilus* regularly roosts with other species in buildings, including bat genera like *Rhinolophus*, which are also known to host high-risk pathogens, these areas may be important hotspots within the wildlife–human urban interface for human exposure to viruses with zoonotic potential [63].

Understanding features of the urban–wildland interface that attract and repel wildlife is important for mitigating human–wildlife conflict that can lead to wildlife mortality. Mass mortality events of bats are generally human-caused, and most species are unable to recover quickly due to low reproductive output [64]. Risk of spillback of zoonotic pathogens from humans to synanthropic bat populations may also exist, with additional negative ramifications for bat health [65]. Bats provide critical ecosystem services, ranging from insect suppression that benefits local agriculture and forestry, to seed dispersal and pollination of many tropical plants, and reducing their mortality directly benefits humans on many levels [15,16]. Thus, our results can help inform the development of methods to reduce bat use of buildings. For example, our study indicates that construction of buildings under four metres in height and with venting to decrease water vapour pressure are likely to reduce bat use. Further, while structural changes are not possible for many existing buildings, emphasis can be placed on sealing entry points so that bats are unable to enter [66]; such actions are especially important for existing tall cement structures.

Ultimately, our results help characterize environmental conditions that can lead to human–bat contact. By identifying microclimate and structural attributes important to building roost selection, we can better understand characteristics that may increase the chance of human–bat interactions, with implications for human–wildlife conflict, pathogen transmission within synanthropic wildlife communities, and potential exposure risk for humans. Efforts to mitigate these interactions are useful in reducing human exposure to pathogens and anthropogenic mortality of bats. Our results show that it can be possible to identify hotspots of human–wildlife interactions in the human–wildland interface, which can help target where measures mitigating contact in urbanizing areas should be applied, especially in the Global South where resources are patchily distributed and may be limited.

## Data Availability

The data are provided in electronic supplementary material [67].
